# Cycle‐generative adversarial network‐based bone suppression imaging for highly accurate markerless motion tracking of lung tumors for cyberknife irradiation therapy

**DOI:** 10.1002/acm2.14212

**Published:** 2023-11-20

**Authors:** Zennosuke Mochizuki, Masahide Saito, Toshihiro Suzuki, Koji Mochizuki, Junichi Hasegawa, Hikaru Nemoto, Kenichiro Satani, Hiroshi Takahashi, Hiroshi Onishi

**Affiliations:** ^1^ Department of Radiology Kasugai‐CyberKnife Rehabilitation Hospital Fuefuki‐city Yamanashi Japan; ^2^ Department of Radiology University of Yamanashi Chuo‐city Yamanashi Japan

**Keywords:** CycleGAN, deep learning, generative adversarial networks (GAN), lung cancer, radiotherapy, stereotactic radiotherapy

## Abstract

**Purpose:**

Lung tumor tracking during stereotactic radiotherapy with the CyberKnife can misrecognize tumor location under conditions where similar patterns exist in the search area. This study aimed to develop a technique for bone signal suppression during kV‐x‐ray imaging.

**Methods:**

Paired CT images were created with or without bony structures using a 4D extended cardiac‐torso phantom (XCAT phantom) in 56 cases. Subsequently, 3020 2D x‐ray images were generated. Images with bone were input into cycle‐consistent adversarial network (CycleGAN) and the bone suppressed images on the XCAT phantom (BSI_phantom_) were created. They were then compared to images without bone using the structural similarity index measure (SSIM) and peak signal‐to‐noise ratio (PSNR). Next, 1000 non‐simulated treatment images from real cases were input into the training model, and bone‐suppressed images of the patient (BSI_patient_) were created. Zero means normalized cross correlation (ZNCC) by template matching between each of the actual treatment images and BSI_patient_ were calculated.

**Results:**

BSI_phantom_ values were compared to their paired images without bone of the XCAT phantom test data; SSIM and PSNR were 0.90 ± 0.06 and 24.54 ± 4.48, respectively. It was visually confirmed that only bone was selectively suppressed without significantly affecting tumor visualization. The ZNCC values of the actual treatment images and BSI_patient_ were 0.763 ± 0.136 and 0.773 ± 0.143, respectively. The BSI_patient_ showed improved recognition accuracy over the actual treatment images.

**Conclusions:**

The proposed bone suppression imaging technique based on CycleGAN improves image recognition, making it possible to achieve highly accurate motion tracking irradiation.

## 1 INTRODUCTION

Stereotactic body radiation therapy (SBRT) has been widely applied as a treatment modality for early stage non‐small cell lung cancer (NSCLC). SBRT combines the techniques of multidirectional irradiation and precise irradiation of lesions to improve local control of tumors confined to the trunk region and reduce adverse events in normal tissue nearby. SBRT is also capable of high efficacy with a single large dose, which cannot be achieved with conventional radiotherapy.^1^ The CyberKnife (CK) System (Accuray Inc., Sunnyvale, CA, USA) integrates a robotic‐positioned linear accelerator, an image‐guided system, and respiratory tracking systems. CK has two respiratory tracking systems: the fiducial‐based target tracking system (FTTS) and Xsight Lung Tracking System (XLTS).^2^ XLTS is a fiducial‐free real‐time tracking system used to irradiate lung tumors that move with respiration.^3^ This facilitates the minimization of radiation exposure to healthy pulmonary tissues, thereby enhancing the efficacy of treatment.^4^, ^5^ This system, called target locating system (TLS), uses a pair of orthogonal x‐ray imagers, which enable real‐time tracking of moving targets by modeling the correlation between the targets and external surrogate light‐emitting diode markers placed on the patient's chest.^6^ With this system, the patient's treatment area is tracked using real‐time imaging, and the error in the irradiated area is less than 1 mm.^7^, ^8^

Generally, sufficient tumor contrast relative to the surrounding tissue in an x‐ray image is essential for accurate soft‐tissue tracking. XLTS performs template matching to match the image density pattern of the tumor region in digitally reconstructed radiography (DRR) with the most similar region in the live x‐ray image. This may affect visibility, especially in cases with overlapping bone. Because the tumor recognition accuracy is not universal for all lesions, the author implemented an approach for bone suppression imaging using a type of general adversarial network (GAN), called cycle‐consistent adversarial network (CycleGAN). In recent years, GANs have shown state‐of‐the‐art performance in many image‐processing tasks.^9^, ^10^ In addition, CycleGAN was proposed to learn translation mappings in the absence of aligned paired images.^10^ Recently, CycleGAN was used to synthesize CT images from cone beam CT (CBCT) images.^11^ However, no research has been conducted to date that incorporates CycleGAN into a CK motion‐tracking algorithm. Therefore, this study aimed to use deep learning to develop a bone suppression imaging technique for kV x‐ray images.

## 2 MATERIALS AND METHODS

**FIGURE 1:**
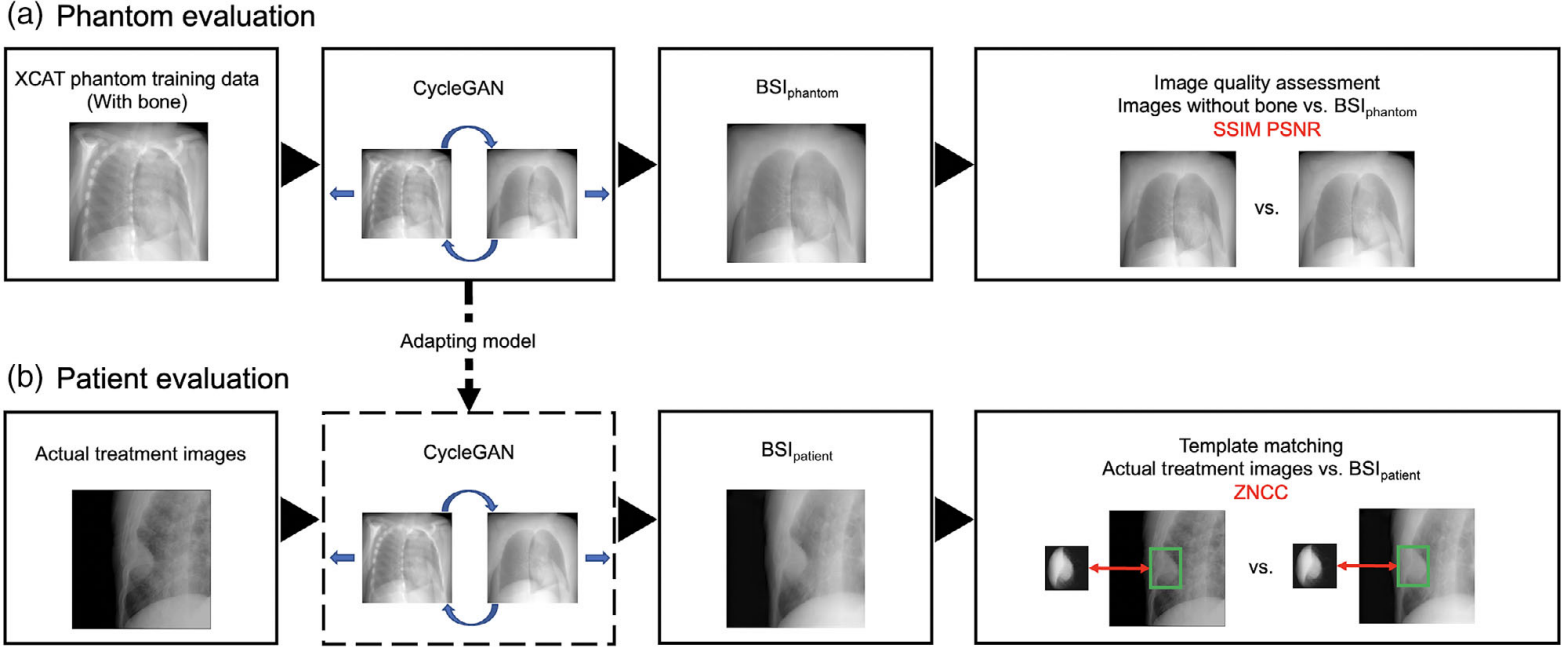
Work‐flow of the bone suppression and evaluation framework.

After the images with bone were input to the trained model to create bone suppressed images, the images generated by CycleGAN (BSI_phantom_) were compared to images without bone. Next, BSI_patient_ were generated from the images used for actual CK treatment, and the effectiveness of deep learning was compared by template matching for each of the actual treatment images and BSI_patient_. The workflow is illustrated in Figure 1.

### 2.1 Data

#### 2.1.1 XCAT phantom (4D extended cardiac‐torso phantom) images

**FIGURE 2:**
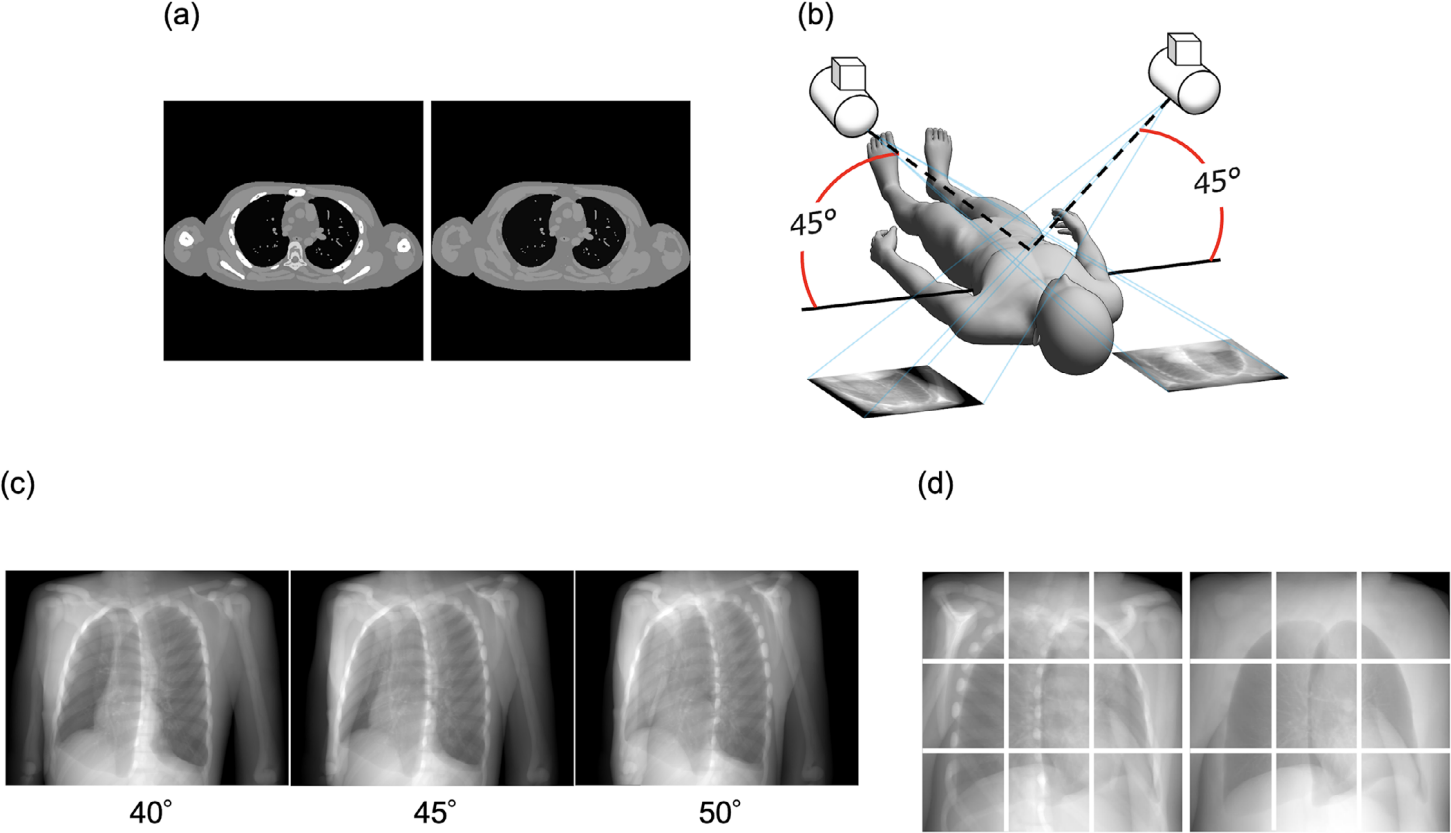
Creation of image datasets with and without bone XCAT phantom. (a): With (L)/without (R) bone generated from XCAT Phantom, (b): Pseudo‐projecting the XCAT phantom, Projections with 5 degree difference, (d): The projected images with (L)/without (R) bone as in (b) were divided into 9 segments.

We first created images from XCAT phantom (Duke University, Durham, North Carolina)^12^, ^13^ to project the images to be used in the deep‐learning datasets. XCAT phantom is a digital anthropomorphic phantom image database that was developed based on the human anatomy database of the National Library of Medicine. Based on patient data and using non‐uniform rational b‐spline surfaces to define the anatomy, the XCAT phantom combines a voxelized approach with a mathematical approach to offer simulated imaging with realistic and detailed organs that remain flexible to allow for anatomical variation and organ deformation.^14^, ^15^, ^16^ In addition, the XCAT phantom is positioned off the center of the image, but patient images in CT images can rotate, shift, or exceed the field of view. Here, we used 56 samples from this phantom, which all differed in age, sex, nationality, height, and weight. The dataset included 56 men and women aged 18−78 years, weight 52−120 kg, height 153−190 cm, and BMI:18‐39 kg/m^2^. The dxcat2 code of the XCATv2 software package was used by turning off the activity unit of the bone to obtain images with and without bony structures, as shown in Figure 2a. For settings, bone size parameter of 1, pixel width and slice width of 0.03 cm, and array size of 512 × 512 were used.

Next, to create 2D CK images based on these images, we used 3D Slicer ver.4.11 software (MIT, Massachusetts, USA) to create 45° images, as shown in Figure 2b. Images at 40° and 50° were created in the same manner in order to increase the number of datasets used, as shown in Figure 2c. The View‐Up vector was set to (x,y,z) = (−1,0,45), Normal vector to (x,y,z) = (any angle,−45,0), and Isocentor position to (x,y,z) = (0,0,0). These were then divided into nine segments, as shown in Figure 2d. This process created a total of 3020 images, of which, data from 340 images were used for testing.

#### 2.1.2 Treatment images

To evaluate the model, data from actual treated patients were required. This was obtained from 50 patients with metastatic lung cancer (tumor size 8–64 mm) and primary lung cancer (tumor size 10–62 mm) undergoing SBRT at our center between February 2020 and January 2022. 40 patients were male and 10 were female. The ages of the patients were between 46 and 93 years (average age, 74.14). All patients were treated with the CK G4 system using 2‐view XLTS (100 kV/200 mA/100 ms).

### 2.2 Optimization and training methods

**FIGURE 3:**
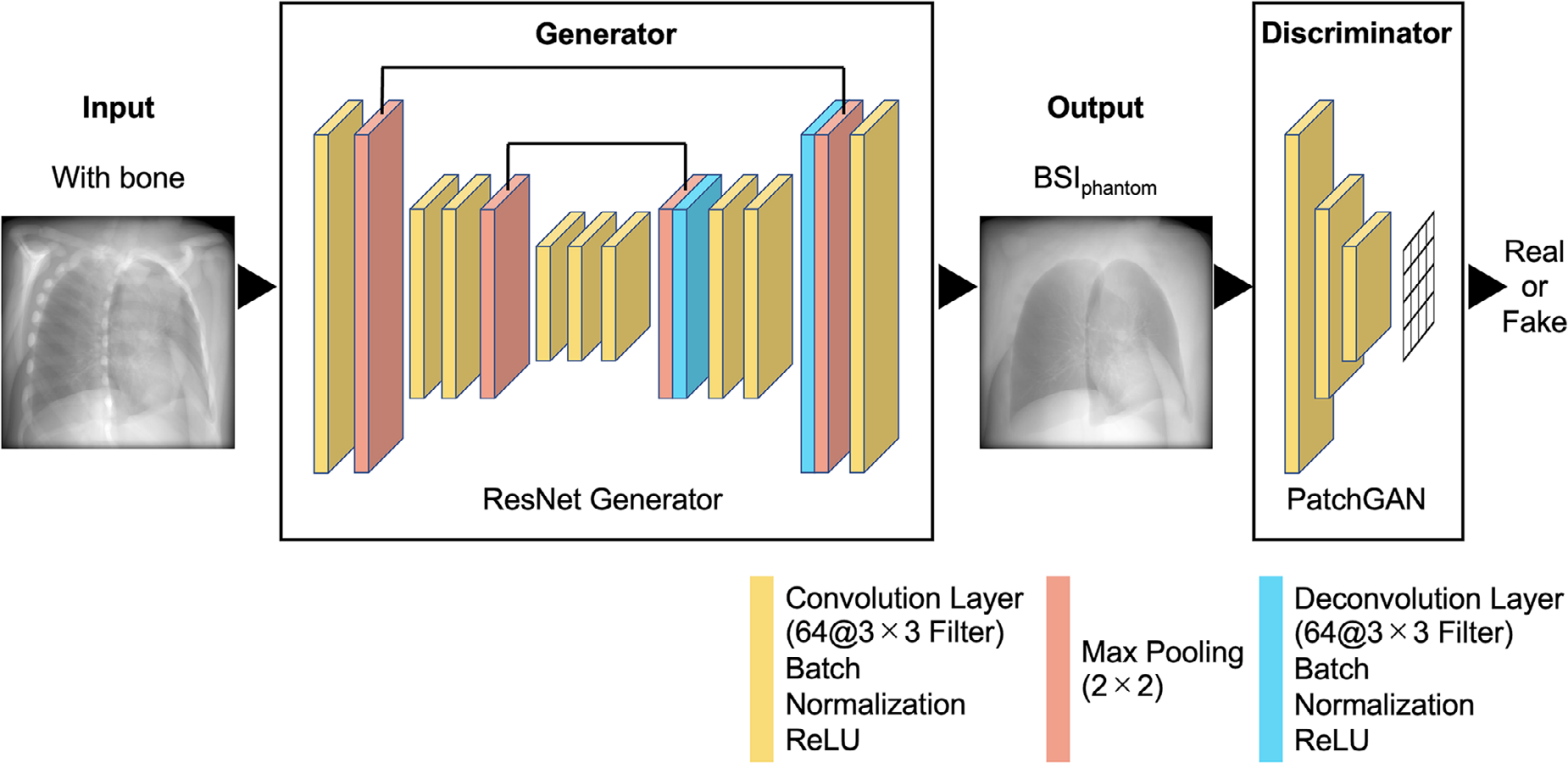
Procedure of CycleGAN. CycleGAN is a method that achieves image transformation by learning domain relationships such as fields and regions between two images.

The training methods and parameters were based on CycleGAN. Similar to pix2pix,^17^ Zhu et al. proposed CycleGAN, a type of image‐generation algorithm using GAN.^10^ Figure 3 shows the CycleGAN procedure. The domain of the images was defined, and images were collected for each domain as training data. The main feature of CycleGAN is that there is no need to prepare a pair of training data. The set of images for each domain is denoted as X and Y, and a generator is prepared to perform X‐to‐Y and Y‐to‐X transformations on them. The default generator architecture of CycleGAN is ResNet, while the default discriminator architecture is a PatchGAN classifier.^17^, ^18^ In addition, two discriminators corresponding to both were prepared. The proposed method enables non‐pairwise image transformations by learning the error (loss) between adversarial loss used in the GAN and the cycle consistency Loss proposed in this study. However, it is shown to be less accurate than pix2pix because of unsupervised learning.

In this study, the algorithm was run on a workstation with an NVIDIA^®^ GeForce^®^ RTX 2080 SUPER™ 8GB GPU. Adam^19^ was used as the optimizer, with β1 = 0.5 and β2 = 0.999. The learning rate was set to 0.0002 for both the generator and discriminator. Both the generator and discriminator were updated once for each iteration, and the training was terminated when each network was considered to have been generated with a certain degree of accuracy.

### 2.3 Evaluation methods

The BSI_phantom_ was compared to images without bone from the test data, and the similarity was calculated. We evaluated the models with two different objective image quality metrics, namely, the structural similarity index (SSIM)^20^ and peak signal‐to‐noise ratio (PSNR).^21^

SSIM is a method to evaluate image quality based on the structural information of an image; it is shown in Equations (1) and (2). Equation (1) was used to obtain the SSIM for each block, and Equation (2) was used to obtain the average SSIM for all blocks.

(1) $$\begin{array}{c} \mathrm{SSIM}\left(x,y\right)=\frac{\left(2\mu_{x}\mu_{y}+C1\right)\left(2\sigma_{\mathrm{xy}}+C_{2}\right)}{\left(\mu_{2}^{x}+\mu_{2}^{y}+C_{1}\right)\left(\sigma_{2}^{x}+\sigma_{2}^{y}+C_{2}\right)} \end{array}$$

(2) $$\mathrm{MSSIM}=1/M\sum_{j=1}^{M}\mathrm{SSIM}\left(x_{j},y_{j}\right)$$

where *x* is one block of the reference image, *y* is one block of the test image, and μ is the average per block of the Gaussian filtered image. Similarly, σ^2^ refers to the variance per block of the Gaussian filtered image: *C*_1_ and *C*_2_ are constants, (0.01 × 255)^2^ and (0.03 × 255)^2^, respectively. *M* is the number of blocks.

PSNR is an index obtained by the ratio of the mean squared error (MSE) of the reference image and the test image to the maximum grayscale value (PS = 255). The PSNR is shown in Equation (3), and the MSE is shown in Equation (4).

(3) $$\mathrm{PSNR}=10\log_{10}\left(\frac{PS^{2}}{\mathrm{MSE}}\right)$$

(4) $$\mathrm{MSE}=\frac{1}{N}\sum_{1}^{N}{\left(x_{i}-y_{i}\right)}^{2}$$

where $x_{i}$ represents the reference image; $y_{i}$ represents the gray scale value of the test image; *N* represents the total number of images.

Next, template matching^22^, ^23^ was performed on each of the actual treatment images and BSI_patient_ to calculate the zero‐mean normalized cross‐correlation (ZNCC).^24^ Therefore, in this study, the search area was the entire image and the goal was to capture the tumor contour. The detection method using template matching is the most basic and determines the location of the target in the input image by comparing the input image with the partial image of the target to be tracked, which was prepared in advance. ZNCC is a measure that uses the average luminance values of the template and input image to compensate for the effects of the lighting environment. The location with the highest value is the target location. In addition, ZNCC is a general measure of image similarity and ranges from −1 to 1, with a value of 1 when the two images are exactly matched. After 1000 actual treatment images were run through CycleGAN to BSI_patient_, template matching was performed on each of the actual treatment images and BSI_patient_ to calculate ZNCC.

### 2.4 Statistical analysis

All statistical analyses were performed using SPSS Statistics for Windows (version 27.0; IBM Corp., Armonk, NY, Version 27.0. Armonk, NY: IBM Corp). Differences between the groups were compared using independent *T*‐tests. Alpha was set to 0.05.

## 3 RESULTS

**FIGURE 4:**
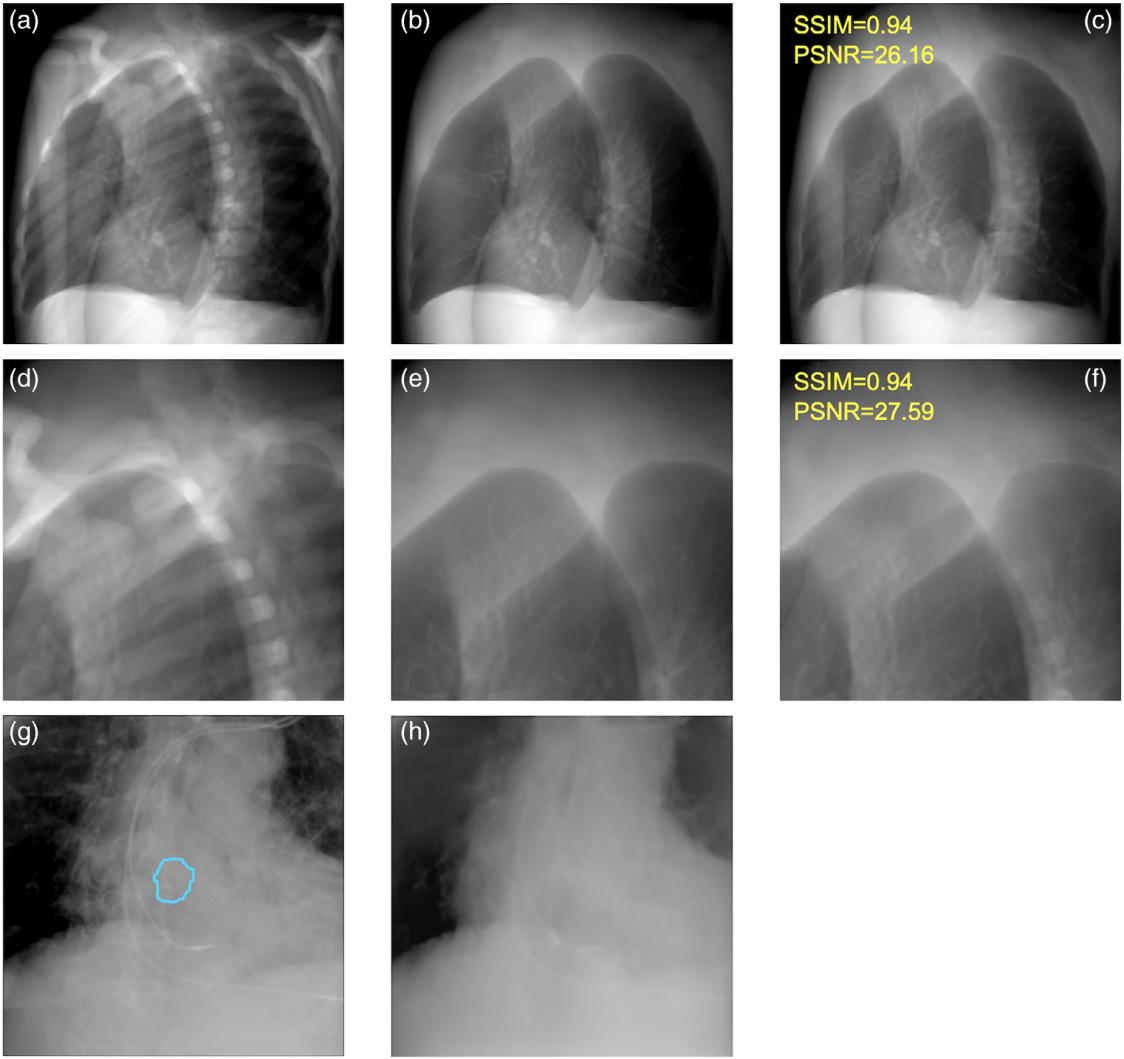
Examples between the test data and BSI. (a): the test data with bone, (b): the test data without bone, (c): BSI_phantom_, (d–f): partially enlarged versions of (a–c), (g): actual treatment images and (h): BSI_patient_, the blue line in the patient data shows the TTV.

Figure 4 shows examples of BSI_phantom_ compared to the test data without bone, and the similarity was calculated. The bone intensity was generally reduced, although some remained intact. The mean of SSIM and PSNR were 0.90 ± 0.06 and 24.54 ± 4.48, respectively, indicating high similarity. Figure 4g,h show examples of image generation using CycleGAN. The blue line indicates a tumor tracking volume (TTV). The bone intensity was suppressed, and tumor contours remained after image generation.

**FIGURE 5:**
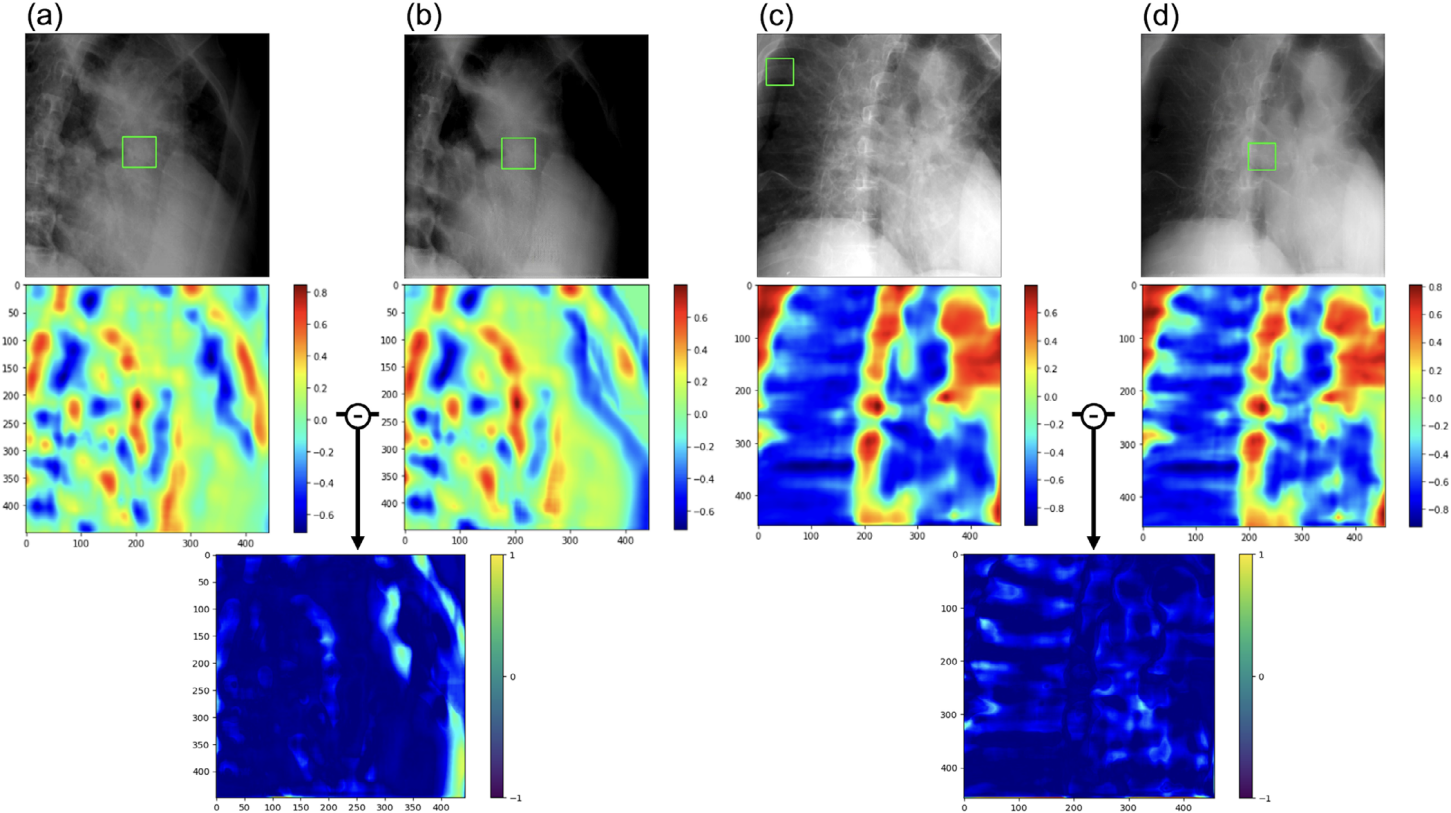
Template matching for each of actual treatment images and BSI_patient_. The upper figure shows (a,c): actual treatment images and (b,d): BSI_patient_. The green box in the patient data shows the region of high similarity in template matching, demarcated by rectangular windows. The middle figure shows heatmaps for the similarity of each image. The bottom figure shows the subtraction images for both pairs.

Next, template matching was performed using the actual treatment images and BSI_patient_, and the ZNCC was calculated for each. Figure 5 shows two examples of template matching. The middle figure shows heatmaps for the similarity of each image. ZNCC is vectorized each cell in the heatmap and calculated the similarity based on its angle. The red color is indicated higher similarity; ZNCC was significantly different and higher for images without bone structures. The heatmap visualizes the model based on these differences.

Table 1 shows the average ± standard deviation of ZNCC for the 767 images that could be detected by template matching. The p‐value was less than 0.05, which was considered statistically significant, indicating that the BSI_patient_ had higher ZNCC than the actual treatment images.

**TABLE 1:** ZNCC was calculated by template matching of actual treatment images and BSI_patient_, respectively.

Image types	Total number of images	Number of images identified correctly (%)	ZNCC
Actual treatment images	1000	767 (76.7)	0.763 ± 0.136^*^
BSI_patient_	1000	800 (80.0)	0.773 ± 0.143^*^

* *p* < 0.05 significantly different compared with the reference.

## 4 DISCUSSION

This is the first report that tried to incorporate CycleGAN into a CK motion‐tracking algorithm, and the promising result is expected to gain worldwide recognition. We propose the use of CycleGAN to learn to suppress the intensity of bony structures on TLS radiographs. Experimental evaluations on the XCAT phantom datasets validated the effectiveness of the framework developed to do this.

Using existing methods, tumors are difficult to detect because the visibility of the tumor in the live image is reduced when the tumor overlaps bony structures, and the image quality is degraded. In this study, deep learning and evaluation were performed using a dataset created from the XCAT phantom to generate bone‐suppression images for the XLTS. This method holds the promise of achieving a high level of accuracy through the training of the model using images with and without bone. However, in this study, actual treatment images were run through CycleGAN to generate images without bone, and there were cases in which bone intensity reduction was difficult. This may be due to the possibility that the images were not recognized as bone, in which case, improved datasets may be required. As a measure to create data sets that suppress bone intensity, image processing techniques such as dual energy subtraction have been used recently in order to improve the detection of pulmonary nodules.^25^ In a study examining the detection of thoracic masses, the use of soft‐tissue images significantly improved the detection of masses.^26^ In addition, a model was constructed to generate soft tissue chest images using GAN, and comparison with test data showed that the generated images had a high degree of similarity, indicating their clinical usefulness.^27^

In addition, there was a discrepancy between the location of the tumor and that indicated by template matching. Because template matching searches for the location where the similarity between the input image and template is maximized, it is difficult to accommodate changes in the appearance of the target, such as rotation. To solve this problem, variable template matching, in which the template is updated when tracking is successful, has been proposed, but there is an accumulation of errors due to background contamination in the template and difficulty in determining the success or failure itself.^28^, ^29^, ^30^

As a limitation, XLTS is used uncertainty values to evaluate detection uncertainty, but the algorithm varies from version to version and the details are not publicly available. Therefore, this was addressed in this study by calculating the similarity using ZNCC.

For images in which tumors could not be recognized using this method, the reason for the failure to recognize the tumors was considered to be poor background removal processing. If the tumor to be recognized is complex and contains considerable noise, noise removal cannot be performed well. Furthermore, recognition becomes impossible owing to the inclusion of noise and the tumor.

Therefore, our future studies will investigate a new recognition method that utilizes new features such as noise reduction and contrast.

## 5 CONCLUSION

In this study, we generated and evaluated CycleGAN images using a dataset created with the XCAT phantom to generate bone suppression images for the XLTS. The proposed bone suppression imaging technique based on CycleGAN improves the image similarity in template matching, making it possible to achieve highly accurate markerless motion tracking irradiation. The possibilities for future studies are included evaluation experiments of the constructed algorithms and their implementation in phantoms on simulated tumors for clinical applications.

## AUTHOR CONTRIBUTIONS

The authors confirm contribution to the paper as follows: study conception and design: Zennosuke Mochizuki, MS, Masahide Saito, PhD; data collection: Zennosuke Mochizuki, MS; analysis and interpretation of results: Zennosuke Mochizuki, MS, Masahide Saito, PhD; draft manuscript preparation: Zennosuke Mochizuki, MS, Masahide Saito, PhD. All authors reviewed the results and approved the final version of the manuscript.

## CONFLICT OF INTEREST STATEMENT

The authors declare no potential conflicts of interest.
